# *Fusarium* spp. associated with *Chenopodium quinoa* crops in Colombia

**DOI:** 10.1038/s41598-022-24908-w

**Published:** 2022-12-02

**Authors:** Ingrid Rocio Fonseca-Guerra, Jhojan Camilo Chiquillo-Pompeyo, Martha Elizabeth Benavides Rozo, Javier Fernando Díaz Ovalle

**Affiliations:** 1 Gestión Ambiental Group, Universidad de Boyacá, 150003 Tunja, Colombia; 2 Quorum Sensing Group, Universidad de Boyacá, 150003 Tunja, Colombia

**Keywords:** Microbiology, Fungi, Fungal biology

## Abstract

Quinoa is a plant commonly-resistance to adverse biotic and abiotic factors. However, this crop can be affected by phytopathogenic fungi. There is a lack of knowledge about the fungi associated with quinoa plants in Colombia. Through morphological and molecular identification in this study were identified four *Fusarium* species associated with quinoa crops: *Fusarium oxysporum*, *Fusarium graminearum*, *Fusarium equiset*i, and *Fusarium culmorum*. For this, we collected samples of panicles, leaf tissue, root tissue, and soil for isolation of different isolates of *Fusarium.* We performed a pathogenicity test of the fungi strains, under greenhouse conditions to evaluate the pathogenicity in seedlings of the Piartal cultivar with two inoculation methods. First inoculating the stem through a nodal wound or second inoculating the abaxial face with a brush. The results indicate the presence of four species with both molecular markers, phylogenetically distributed in these groups. The four species turned out to be pathogenic but with different degrees of virulence with significant differences between *F. graminearum* and *F. oxysporum* depending on the inoculation method. This is the first report on the presence of *Fusarium* species isolated from Quinoa in Colombia.

## Introduction

*Fusarium*, a cosmopolitan group of fungi, encompasses around 300 phylogenetically distinct species that can present groupings called species complexes, of which 23 complexes have been described^1^. In recent years this fungus has received considerable attention because it can affect a large number of crops worldwide, leaving devastating annual economic losses^2^. Additionally, in this genus, more than 70 species are characterized as opportunistic pathogens, which can generate grave infections in immunodeficiency patients^3^. The mycotoxins can cause serious diseases in animals and humans and the presence of mycotoxigenic strains in foods is alarming because more than one type of these secondary metabolites can be found in different food sources^4^. This genus is so crucial in the agricultural and food industry because more than a hundred economic interest plants, proposed at least around 80, are attacked by some species of *Fusarium*^5^.

The multihost capacity of this fungus, and its ability to infect different tissues and organs such as roots, stems, leaves, fruits or seeds cause a decrease in yield and a decrease in the quality of agricultural by-products^6^. Diseases caused by *Fusarium* present a challenge in diagnosis and control, specifically with soil-transmitted species such as *Fusarium oxysporum*^7^*.* Therefore, the timely identification of these phytopathogens allows the development of action plans to treat and prevent the spread of this pest at an appropriate time, being the essential molecular tool to give a solid response to this problem. Some molecular markers such as translation elongation factor (EF1-α), β-tubulin, calmodulin, intergenic spacer region (IGS), and internal transcribed spacer (ITS) are used to identify, classify and differentiate *Fusarium* species^8^.

Although this genus is recognized worldwide as an important pathogen, it is necessary to mention that some isolates of this genus are of great biotechnological importance due to their ability to produce enzymes and secondary metabolites used in various industrial processes^8,9^.

The FAO named quinoa as one of the XXI century grains. This pseudocereal is rich in vitamins, minerals, proteins, and it has a large percentage of essential amino acids, lipids, and carbohydrates. In this sense, its grain can help solve nutritional problems. Besides, quinoa is ideal for groups with special requirements, among which are children, elderly adults, people with obesity, dyslipidemia or diabetes^10,11^. Additionally, *C. quinoa* has exceptional nutritional qualities, wide genetic diversity and a great adaptation to different biotic and abiotic stress conditions, such as variable temperatures, high salt concentrations, water deficit, acidic or alkaline soils, among other^12,13^. Additionally, quinoa leaves and grains exhibit excellent potential for application in the food and pharmaceutical industries because they have an appreciable phenolic content, a higher content of the antinutrient saponin in the grain, and more nitrates and oxalates in the leaves^14^.

Despite the broad resistance reported in this crop, different varieties are often affected by phytopathogens, mainly of fungal origin. With the highest frequency of the *Peronospora* genus, the etiological agent of downy mildew is the most recognized pathology in this crop. Its pathogen corresponds to the pseudo-fungi of the Oomycetes group, and this fungal disease has been reported in South American countries such as Chile, Peru, Bolivia, Ecuador, and Colombia^15^. The study of other phytopathogenic fungi that affect this pseudocereal is limited, we found principally reports of the *Ascochyta* spp., *Cercospora* spp., *Colletotrichum* spp., *Phoma* spp., *Sclerotinia* sp., *Rhizoctonia* sp. and *Fusarium* spp.^16,17^. Regarding bacteria, the symptoms in foliar tissue in *C. quinoa* caused by *Pseudomonas syringae* for Colombia have been described for the first time by our research group^18^.

The present study focuses on the identification of *Fusarium* spp. associated with quinoa crops in Boyacá, Colombia. We use ITS and EF1-α molecular markers for the PCR. The strains were isolated from quinoa crop leaves, roots, and rhizospheric soil. Additionally, we describe the morphological features and the pathogenic potential of these isolates.

## Results

### Presence of *Fusarium* spp

We found a significant presence of *Fusarium spp.* in most of the sampled localities (Table 1) and we observed in the field that this genus is mainly associated with young plants, predominantly over other genres. We managed to isolate *Fusarium culmorum* from roots and leaves, *Fusarium graminearum* from leaves *Fusarium equiseti* from soil and leaves and *F. oxysporum* from rhizospheric soil, roots, stems and leaves the latter presented the highest frequency of isolation.

**Table 1:** Presence of *Fusarium* spp. in the study zone.

Municipality zone	Cultivar	*F. graminearum*	*F. oxysporum*	*F. culmorum*	*F. equiseti*	*Fusarium sp.*
Tunja Centro	Blanca de Jericó	X	X	X		
Tunja Porvenir	Blanca de Jericó	X	X		X	
Cómbita San Martín	Boyacá Real		X			X-
Cómbita San Onofre	Boyacá Real					X
Siachoque Tocavita	Blanca de Jericó		X	X	X	
Siachoque Guaticha	Piartal		X	X	X	
Tuta Hacienda	Blanca de Jericó	X	X	X	X	
Tuta Aguablanca	Blanca de Jericó	X	X	X	X	
Soracá Otrolado	Blanca de Jericó	X	X		X	
Tibasosa Patanegra	Piartal	X	X	X		
Oicatá Centro						
Chivatá San Francisco	Blanca de Jericó		X		X	
Sotaquirá Cortadero	Piartal		X	X		

### Morphological characterization of *Fusarium* spp.

**Figure 1:**
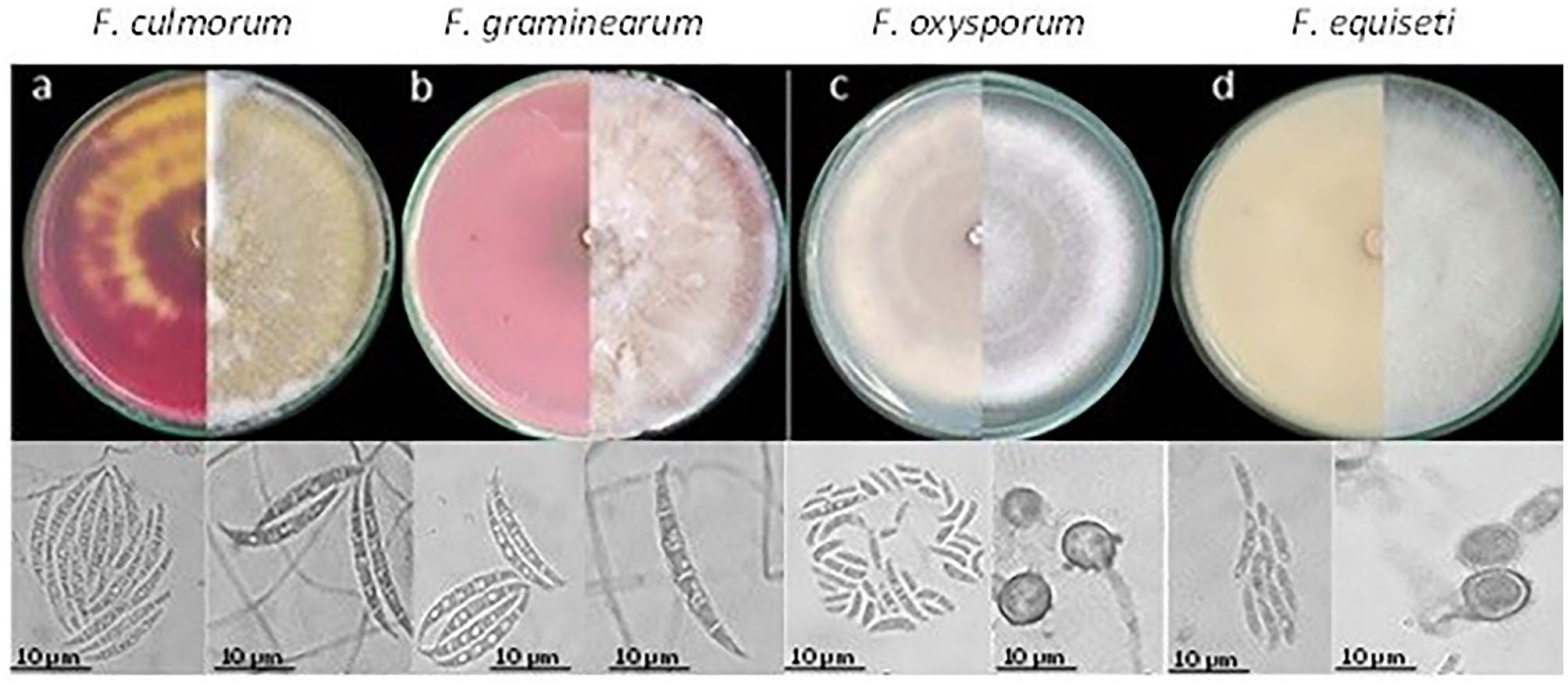
Macro and microscopic characteristics of the *Fusarium* complex associated with quinoa crops. On top the growth of the colonies of the 4 species *F. culmorum*, *F. graminearum F. oxysporum* and *F. equiseti* in PDA culture media after 7 days of incubation at 28 °C, at back side (right) and front side (left). Down the microscopic structures obtained after 14 days of incubation in SNA at 25 °C, and 12-h light/dark photoperiod. Scale of 40 × magnification.

The four species found in this study corresponding to *F. oxysporum*, *F. graminearum*, *F. equiseti* and *F. culmorum*; the description of the colonies was carried out on PDA agar, while the description of the microscopic characteristics was carried out on carnation agar and SNA agar (Fig. 1).

*F. culmorum* has velvety or cottony colonies, presence of aerial mycelium, white superficial color and in the background, it presents a pale brown–brown coloration. Microscopic structures such as septate and hyaline hyphae were observed. The presence of macroconidia is uniform and thick in shape, not so long and slightly curved with four to six septa per cell (Fig. 1a)^19,20^.

*F. graminearum* were fast-growing colonies on PDA agar, cottony texture, and presence of dense and diffuse aerial mycelium white on the front, but with reddish pigmentation on the back of the box. On the other hand, structures such as septate hyphae with globose and intercalary chlamydospores were observed (Fig. 1b)^19,20^.

*F. oxysporum* presents colonies with a cottony texture, slightly flat with white edges and a slightly red/carmine red center that when the colony ages it turns to a pyonnotal form;. Micromorphology revealed hyaline and septate hyphae with the presence of short mono phialides and chain chlamydospores arranged terminally or intercalated. (Fig. 1c)^19,20^.

*F. equiseti* presents macroscopic characteristics such as fast-growing colonies on PDA, abundant aerial mycelium, cottony and white that turns pale brown to grayish over time. were distinctly curved, with five to seven septa and hyaline, septate, and microsiphoned hyphae were observed (Fig. 1d)^19,20^.

### Molecular phylogeny

**Figure 2:**
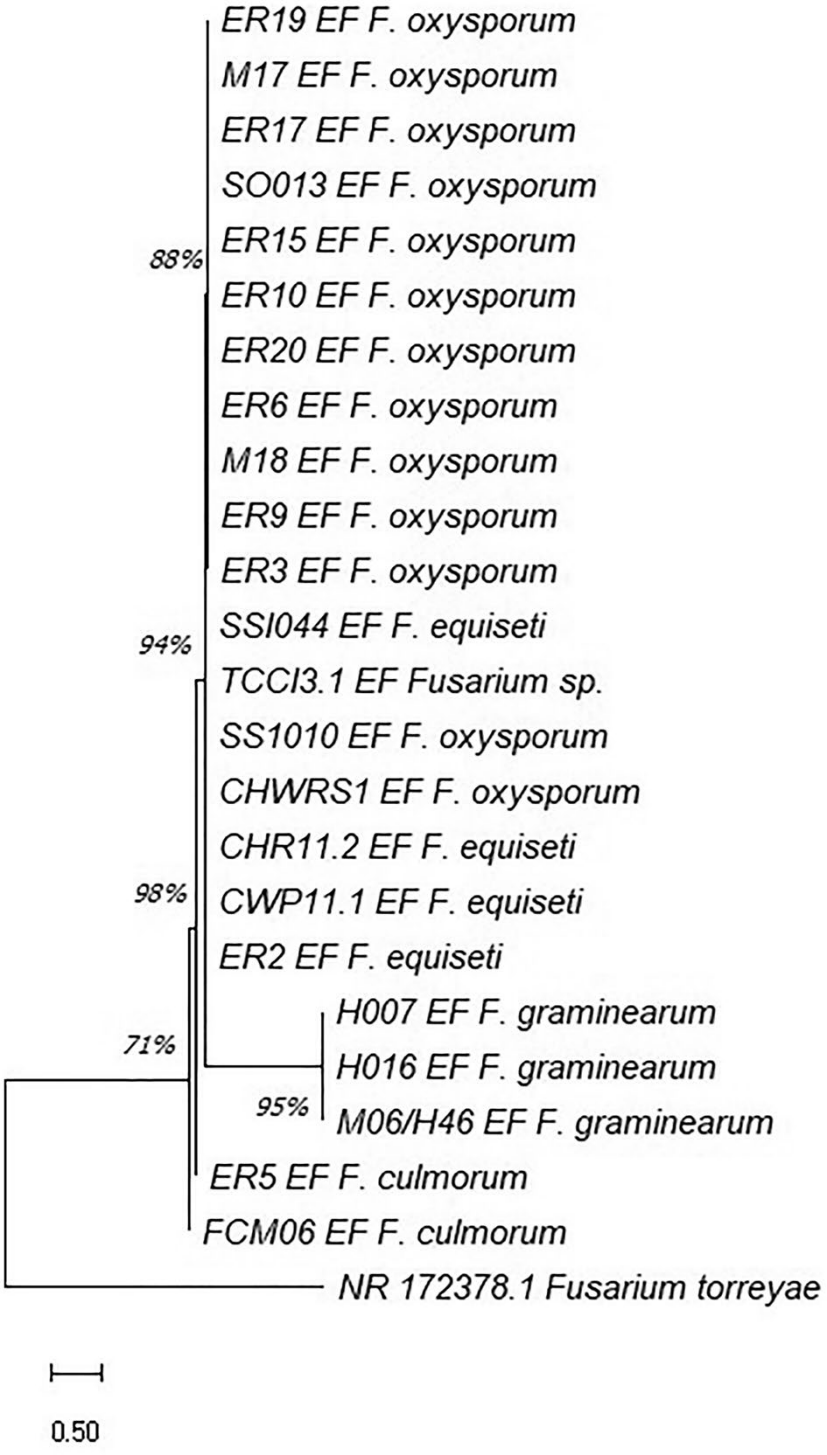
Phylogenetic tree of *Fusarium* isolates in quinoa, based on neighbor-joining analysis of the EF1-α gene. Bootstrap values are from a bootstrap test of 1000 replicates. NR_172378.1 corresponds to the *F. torreyae* outgroup.

**Figure 3:**
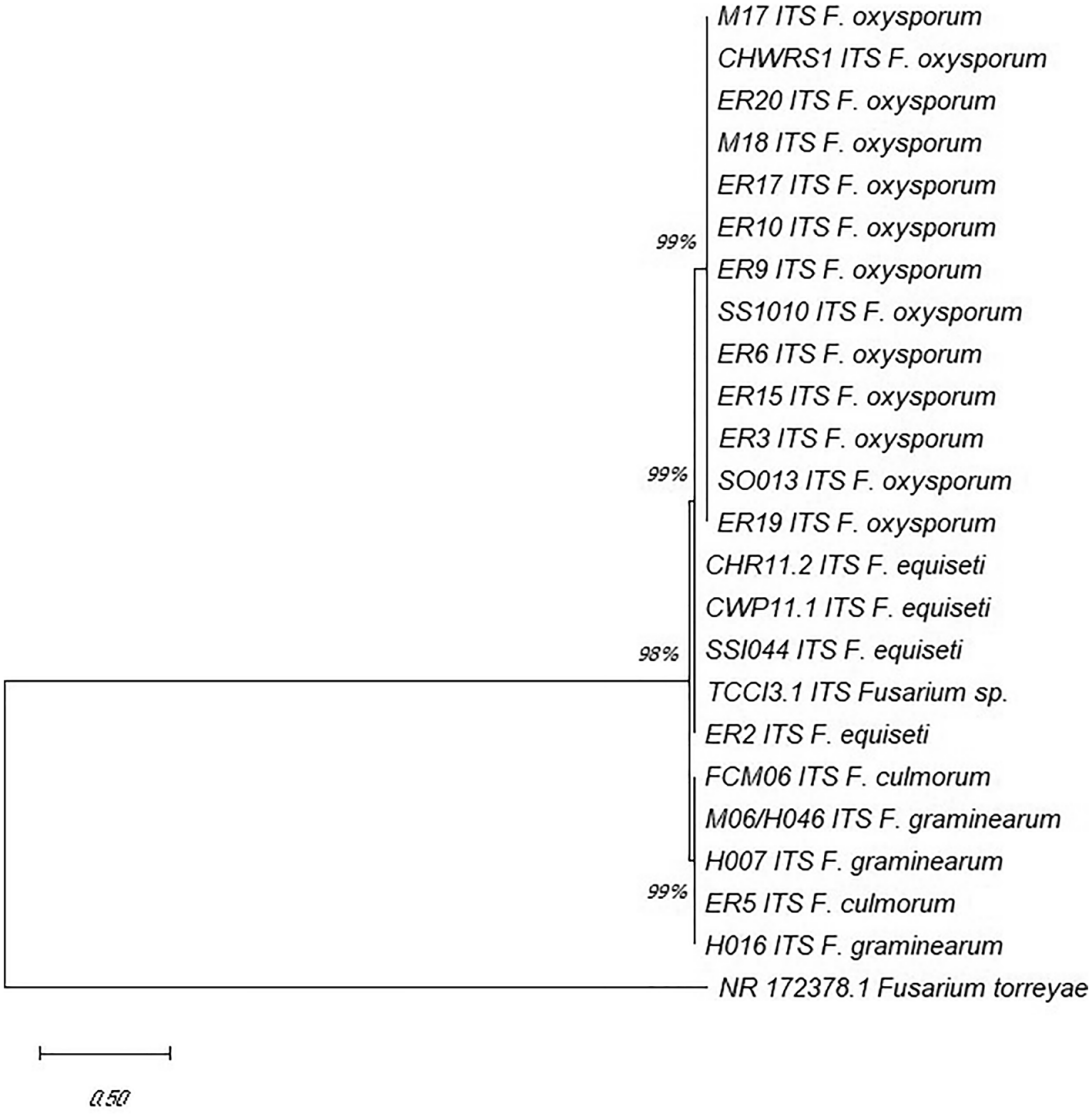
Phylogenetic tree of *Fusarium* isolates in quinoa, based on neighbor-joining analysis of the ITS gene. Bootstrap values are from a bootstrap test of 1000 replicates. OM117609.1 corresponds to *F. torreyae* outgroup.

In the present study, 23 sequences for the ITS region and 23 for the EF-1α gene were analyzed for phylogenetic tree constructions (Table 2). The BLASTN analysis of the isolates confirmed the identity of the four *Fusarium* species, with a reliability level of 99–100% with both molecular markers. Two neighbor-joining trees based on the EF-1α (Fig. 2) and ITS regions (Fig. 3) were constructed. As an outgroup NR_172378.1 (EF-1α) and OM117609.1 (ITS) accession numbers corresponding to *F. torreyae* were selected from GenBank^21^. As shown in Figs. 2 and 3, all isolates were clearly classified into four species, including *F. oxysporum, F. graminearum, F. equiseti* and *F. culmorum*.

**Table 2:** List of *Fusarium* complex isolates sequenced in the present study and their GenBank information together with their relatives to build a phylogenetic tree.

Isolates/collection registration code*	Origin	Specie	GenBank Accession Number	
			ITS	EF
ER2/AM-0156	Siachoque/root	*F. equiseti*	OK509819	OL739195
ER5/AM-0159	Siachoque/root	*F. culmorum*	OL703313	OM363262
ER6/AM-0160	Siachoque/root	*F. oxysporum*	OK509820	OL689620
SSI044/AM-202	Siachoque/soil	*F. equiseti*	OK509836	OL739198
SS1010/AM-224	Siachoque/soil	*F. oxysporum*	OL703314	OM363263
ER9/AM-0163	Soracá/root	*F. oxysporum*	OK509821	OL689621
ER10/AM-0164	Soracá/root	*F. oxysporum*	OK509822	OL689622
SO013/AM-0182	Soracá/soil	*F. oxysporum*	OK509832	OL689627
ER15/AM-0169	Tuta/root	*F. oxysporum*	OK509823	OL689623
ER17/AM-0171	Tuta/root	*F. oxysporum*	OK509824	OL689624
M18/AM-0208	Tuta/leaf	*F. oxysporum*	OK509837	OL689629
ER19/AM-0173	Tibasosa/leaf	*F. oxysporum*	OK509826	OL689625
ER20/AM-0174	Tibasosa/root	*F. oxysporum*	OK509827	OL689626
FCM06/AM-188	Tibasosa/leaf	*F. culmorum*	OK509833	OL739196
M17/AM-0198	Tunja/leaf	*F. oxysporum*	OK509834	OL689628
M06-H046/AM-200	Tunja/leaf	*F. graminearum*	OK509835	OL739203
CHR11.2/AM-0197	Chivatá/leaf	*F. equiseti*	OL703315	OL739197
CWP11.1/AM-0194	Chivatá/leaf	*F. equiseti*	OK509838	OL739199
CHWRS1/AM-0155	Chivatá/stem	*F. oxysporum*	OL703317	OM363264
TCCI3.1/AM-0213	Cómbita/leaf	*Fusarium sp.*	OM337871	OM363265
H007/AM-0206	Tunja/leaf	*F. graminearum*	OL703318	OL739201
H016/AM-0207	Tunja/leaf	*F. graminearum*	OL703319	OL739202
ER3/AM-0157	Tunja/leaf	*F. oxysporum*	OL703320	OL739200

The asterisks indicate the registration code of the isolates in the “Colección de Hongos y Microorganismos de la Universidad de Boyacá '', UBCHM. The markers used did not allow the identification of the species level of the isolate TCCI 3.1/AM-0213.

### Koch's postulates

**Figure 4:**
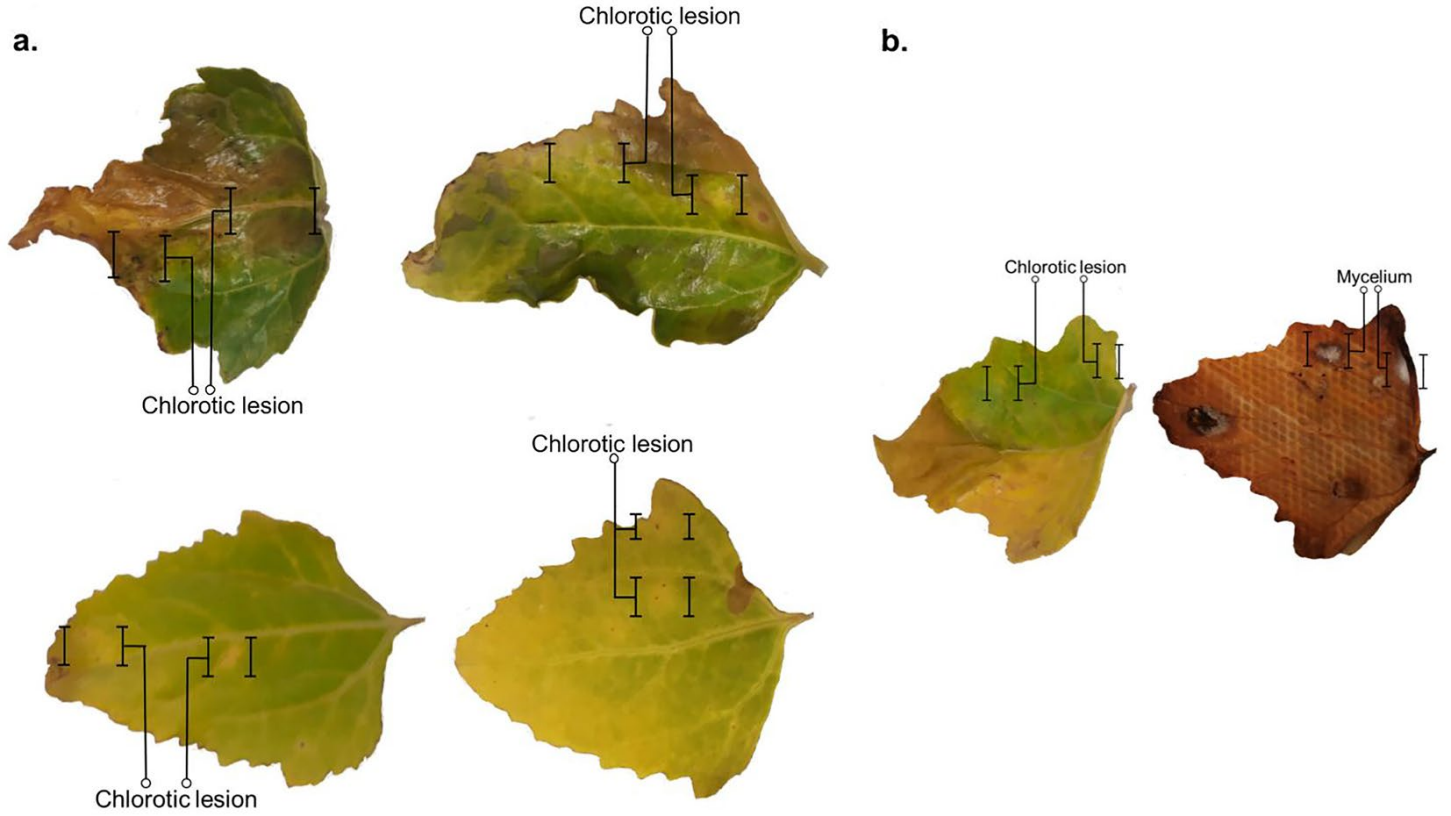
Lesions observed in the field crops that presented growth of *Fusarium* spp. (**a**) Leaves collected with different degrees of affectation. (**b**) The lesions corresponding to *Fusarium* spp. were determined by the growth of the mycelium, after seven days of incubation.

From leaves collected in field crops, the *Fusarium* spp. strains were isolated and purified from the mycelia developed on chlorotic and necrotic lesions as shown in Fig. 4. Thus, this genus was rarely found alone; we find that *Fusarium* spp. is usually associated with *Alternaria* sp. causing round, brown spots with concentric rings in the leaves and *Cladosporium* sp. causing small leaf spots (less than 1 mm) in quinoa plants of the studied area (data no show), in some cases, the presence of *P. syringae*, which causes wilting and bacterial leaf spot, we have also reported^18^.

**Figure 5:**
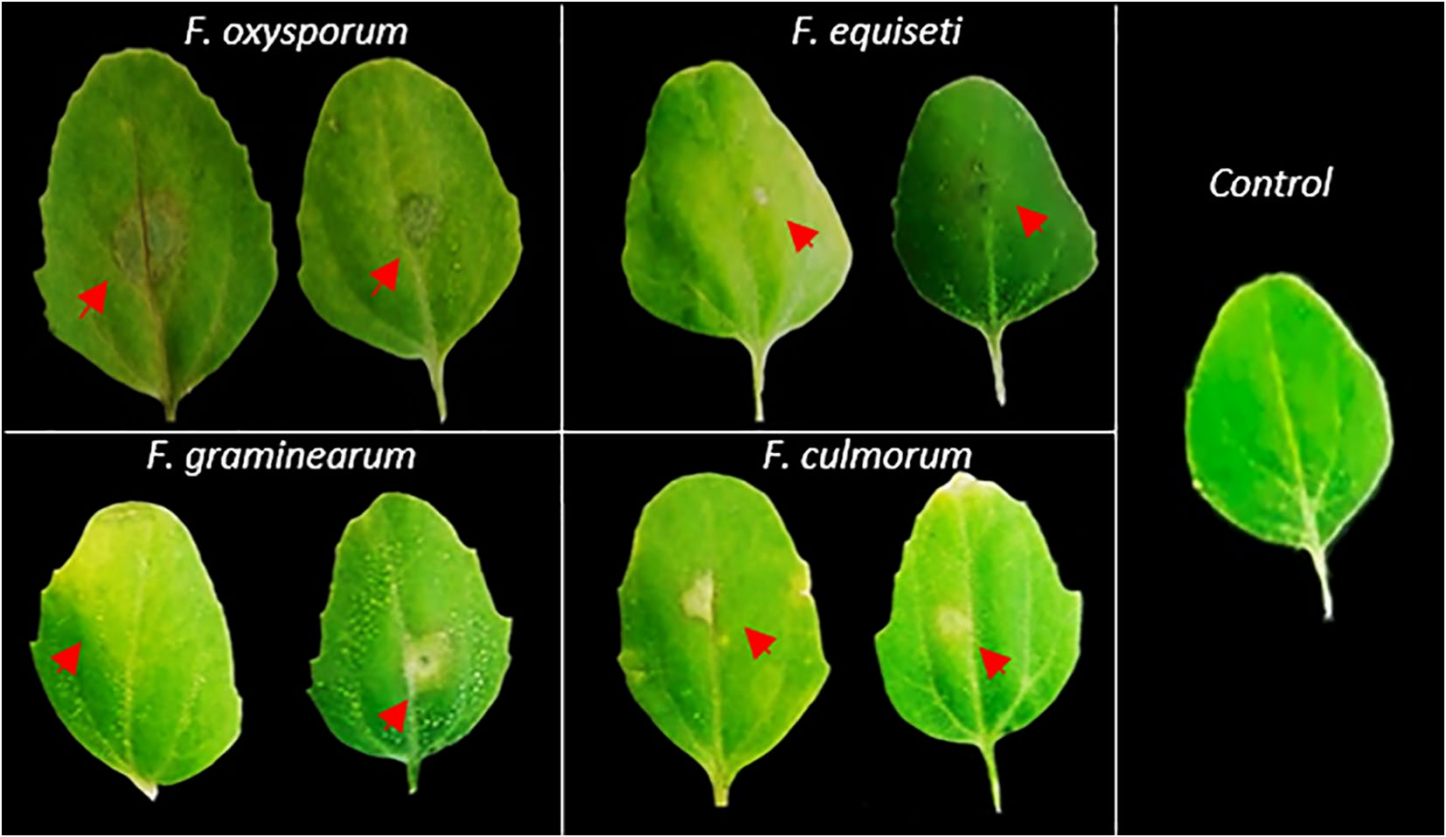
Lesions induced by *Fusarium* spp. observed in leaves inoculated with isolated mycelia. Chlorotic lesions are observed on leaves incubated in a humid chamber. The photographic record corresponds to after five days post inoculation.

We apply Koch's postulates and pathogenicity test to confirm the symptoms and the pathogenic character of the isolated. First applying the protocol proposed by Schuck et al.^22^ we observed that the leaves develop chlorotic spots very similar to those observed in the field (Fig. 5)^22^.

### Pathogenicity test

We evaluated the pathogenicity of *Fusarium* species through two methods. The first method consisted of inoculating the leaves with a brush on the abaxial side of the leaves (brush inoculation) in a high humidity microenvironment (see methods). For the second method (wound inoculation), the inoculum was applied through a wound in the nodal stem; once the stem was inoculated, the wound was covered with a moist gauze and parafilm.

**Figure 6:**
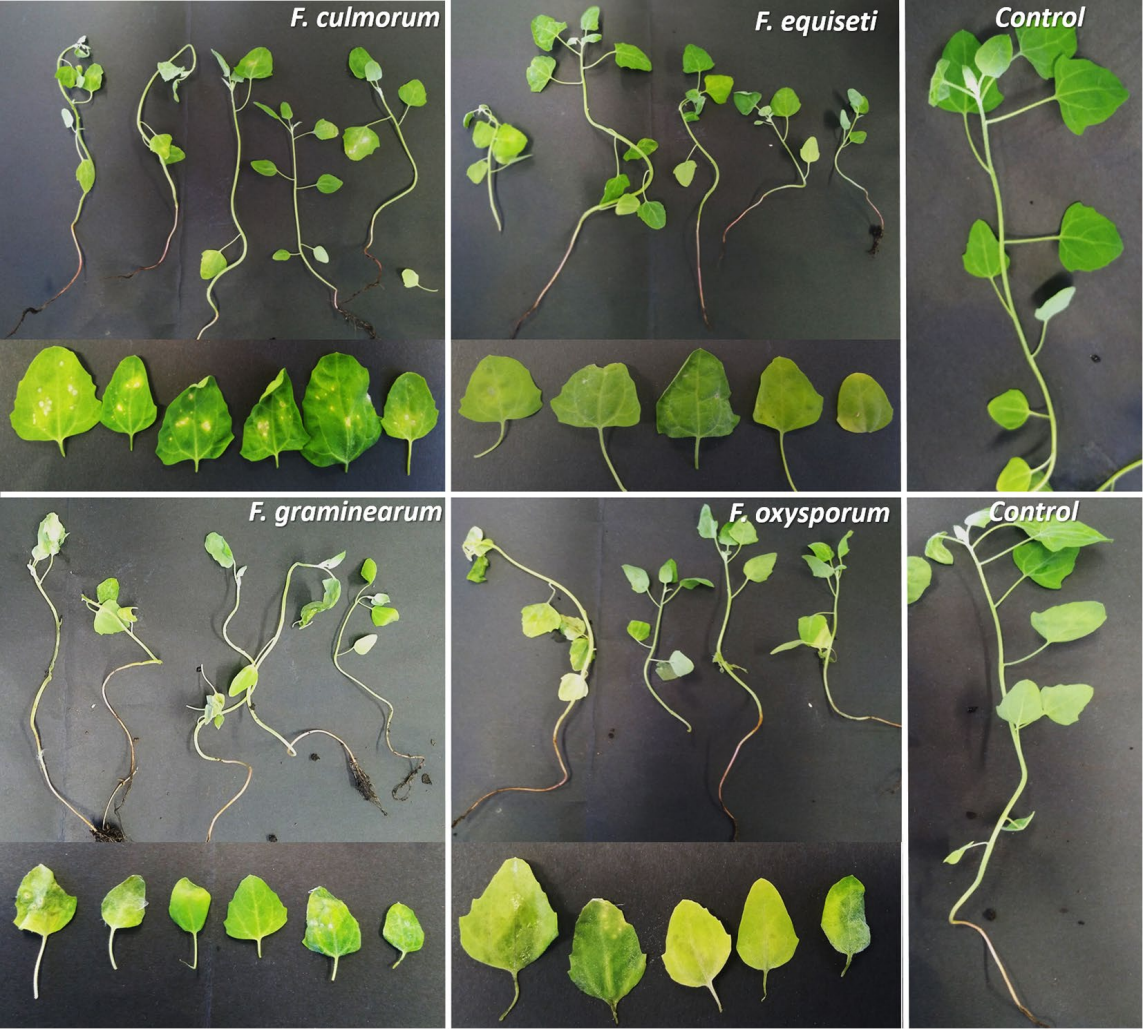
Symptomatology induced by *F. culmorum*, *F. equiseti*, *F. graminearum* and *F. oxysporum* in Piartal cv seedlings inoculated by brush method. The symptomatology is observed on the plant (above) and its detail in leaves (below). The control corresponds to plants without fungal inoculum. Symptoms were observed after 10 days of inoculation.

**Figure 7:**
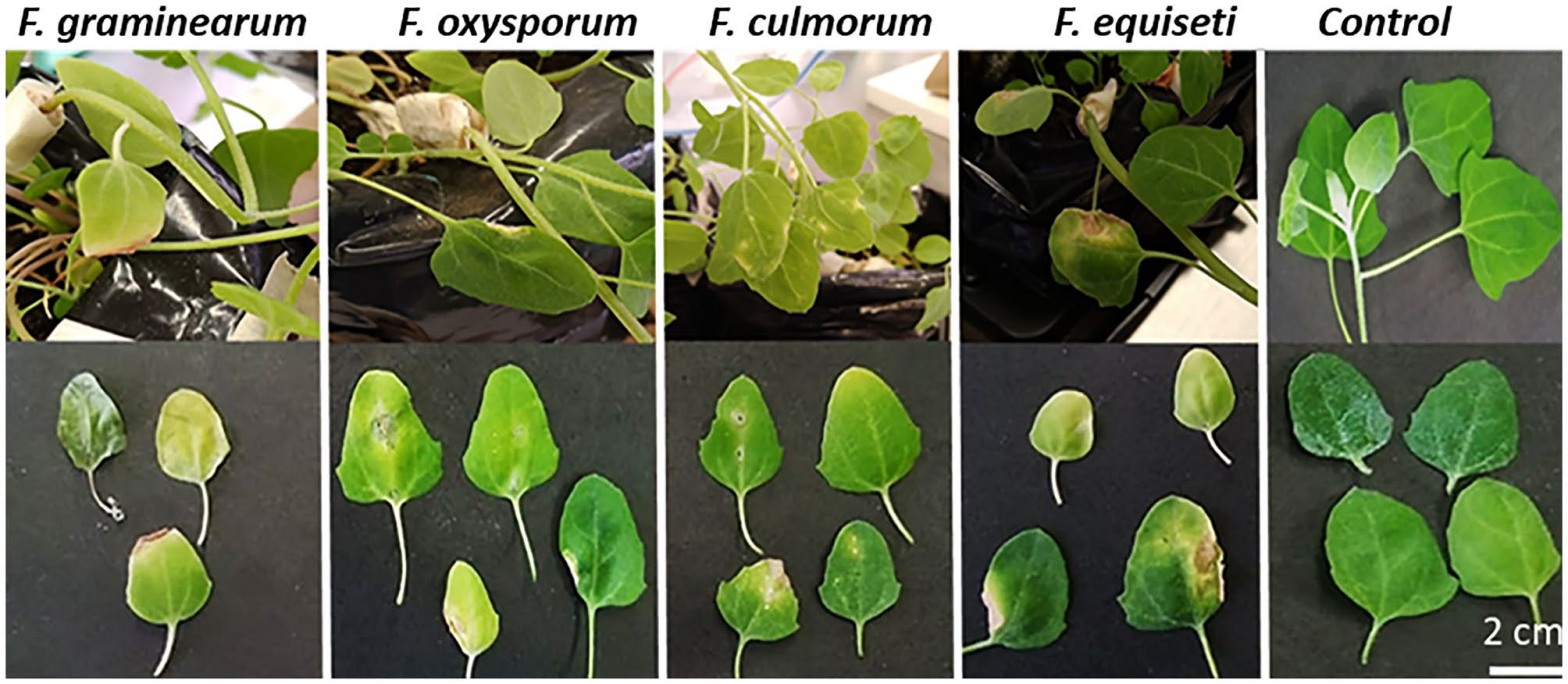
Symptomatology induced by *F. graminearum*, *F. oxysporum*, *F. culmorum* and *F. equiseti* in Piartal cv seedlings inoculated by wound method. The symptomatology is observed on the plant (above) and its detail in leaves (below). The control corresponds to leaves without fungal inoculum. Symptoms were observed after 90 days of inoculation.

We observed symptoms with both methods. However, in the brush inoculation method, the development of symptoms was evident ten days after inoculation (Fig. 6), while in the wound inoculation method (Fig. 7), their appearance took up at least 90 days.

**Figure 8:**
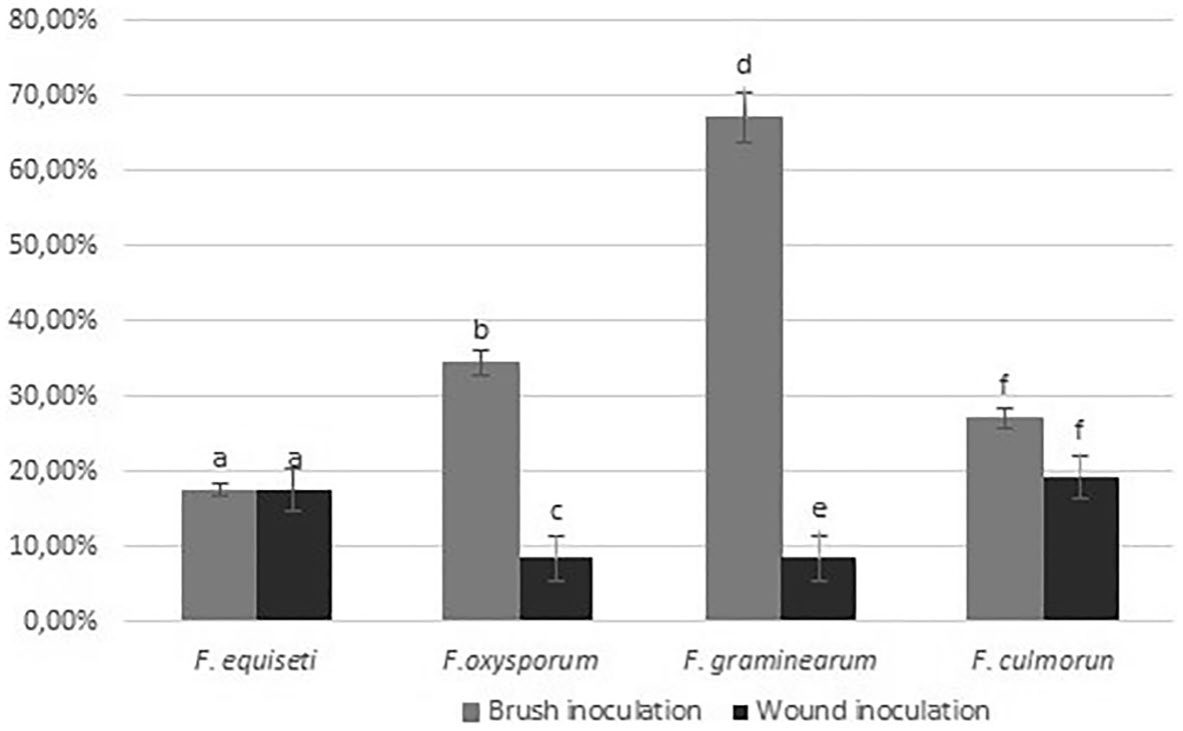
Disease Incidence of *F. equiseti, F. oxysporum*, *F. graminearum*, *F. culmorum* in quinoa seedlings. The data are expressed as the mean with confidence interval using Student's T-test (*p* < 0.05). Letters indicated statistically significant differences between brush and wound inoculation, (**a**) for *F. equiseti*, (**b**,**c**) for *F. oxysporum*, (**d,e**) for *F. graminearum* and (**f**) for *F. culmorum*.

We calculated the disease incidence for each species in the two methods. Our results suggest that the most virulent strain is *F. graminearum* (reaching up to 65% of incidence) and *F. oxysporum* (reaching 38%); however, for both species, the development of the disease depended on the inoculation method. While for *F. culmorum* (${\overline{\text{x}}}$ 25%) and *F. equiseti* (${\overline{\text{x}}}$ 18%), this was not relevant according to the statistical analysis (*p* < 0.05) (Fig. 8).

In plants, our results showed that the disease symptoms in Piartal cultivar corresponded to yellowing mainly induced by *F. graminearum* and *F. oxysporum*. *F. culmorum* induced mottling and was the most virulent species for the wound inoculation method. *F. equiseti* and *F. graminearum* induced leaf spots or necrotic lesions). We determined that although *F. equiseti* is the least virulent species, all four species can induce some foliar damage to the plant.

**Figure 9:**
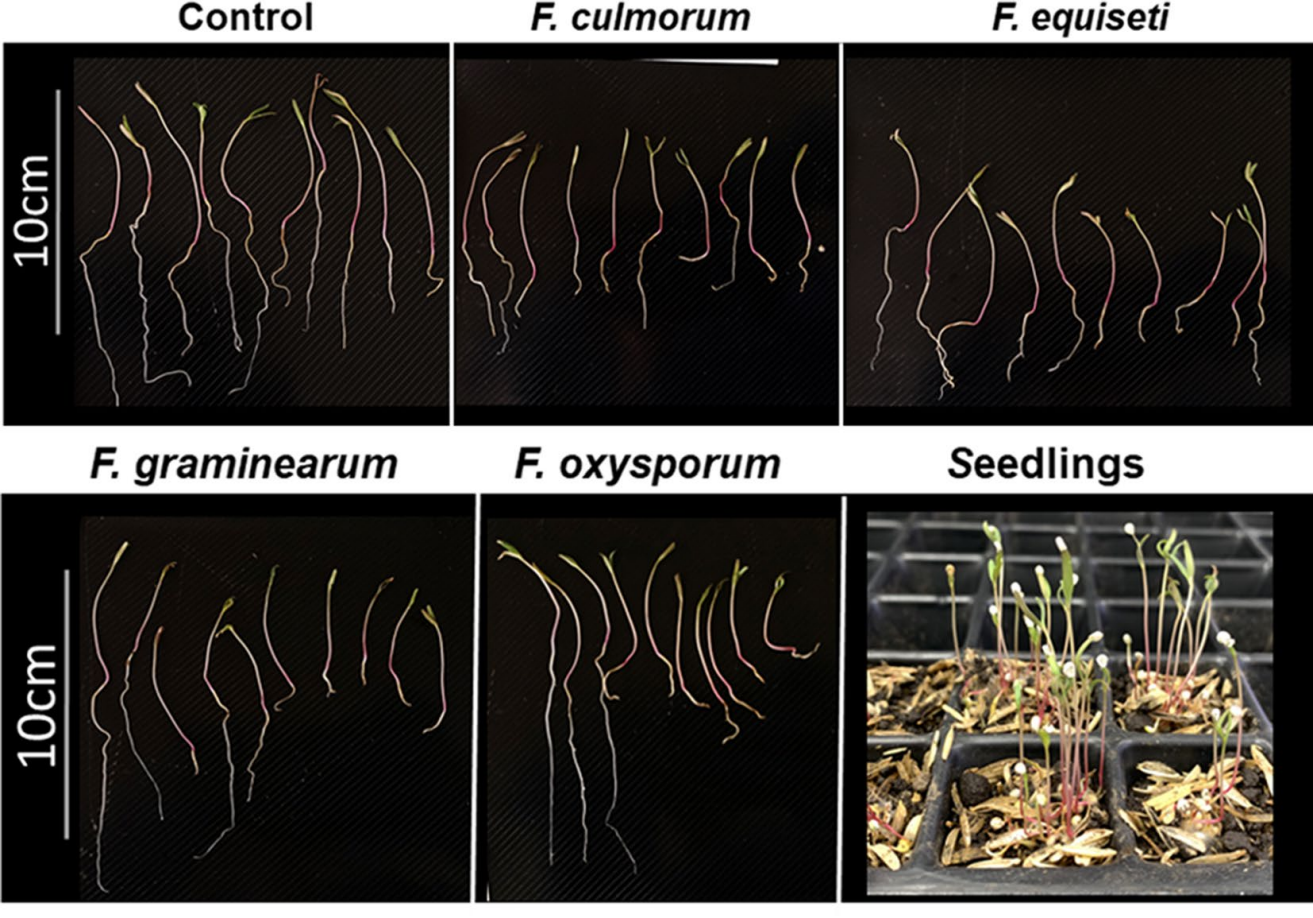
Affectation of root development induced by *Fusarium* spp. in 10-day-old quinoa seedlings. Development of seedlings of the Piartal cultivar in soils inoculated with *F. culmorum*, *F. equiseti*, *F. graminearum* and *F. oxysporum*, under greenhouse conditions. It is observed that the root development was affected by the treatments.

**Figure 10:**
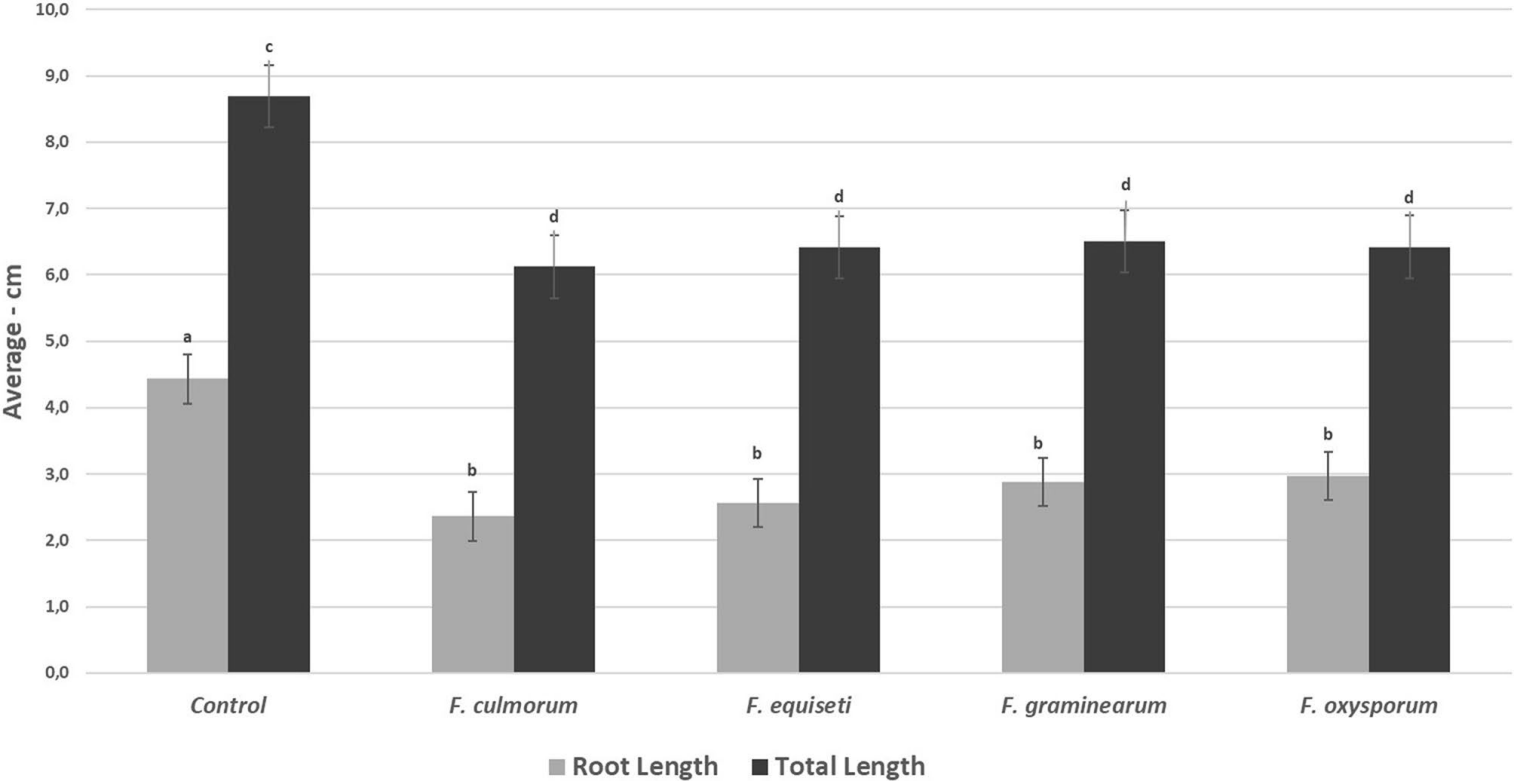
Average total length and root length of seedlings inoculated with *Fusarium* spp. under greenhouse conditions. The letters indicate significant differences between the control and the treatments (a, b) for root length and (c, d) for total length. The data are expressed as the mean with confidence interval using Student's T-test (*p* < 0.05).

Regarding soil inoculation, our results indicate that the four species found affect the root development of seedlings (Fig. 9). Although we found significant differences between the control and the inoculated plants, we did not observe these differences between the four treatments. Additionally, on the tenth day after inoculation, we did not observe chlorosis in the seedlings but a noticeably shorter length (Fig. 10). Likewise, significant differences were observed in the average fresh mass (0.110 gr) with respect to the inocula, but not between the inoculated seedlings (*F. equiseti* 0.097gr, *F. culmorum* 0.98gr, *F. graminearum* 0.099gr, *F. oxysporum* 0.102gr).

## Discussion

In our research, *Fusarium* was a widely distributed genus since it was found in almost all the study areas (only in Oicatá locality no presence was found), which implies a high presence of this genus for environmental conditions of the region. We consider it necessary to evaluate the impact of this fungus on quinoa crops since this fungal genus included several phytopathogenic species that colonize the roots of plants, leading to a decrease in crop yields and developing significant economic losses^23^. Knowledge of the microbiota associated with quinoa is still limited; even so previously, there have been reports of fungi related to the crop of agricultural importance. González-Teuber et al*.* (2017) report in the Atacama Desert, a dominant phylum Ascomycota at a root level in quinoa plants, with the most prevalent genera being *Penicillium, Phoma*, and *Fusarium*, while *Alternaria* spp., *Rhinocladiella* spp., *Cadophora* spp., *Bartalinia* spp., *Coniochaeta* spp., *Neonectria* spp., *Sarocladium* spp. and *Plectosphaerella* spp. were found in smaller proportions, similar to our study^24^. In this sense, Aletaha et al*.* (2018) collected about 52 samples of the subfamily Chenopodioideae in three Iran regions to isolate fungi endophytic roots; the authors found that from 192 samples, the most prevalent microorganism was *Fusarium*. Another fungus also identified corresponded to *Alternaria, Bipolaris, Chaetomium, Cladosporium, Curvularia, Embellisia, Macrophomina, Ulocladium Acremonium, Aspergillus, Penicillium genus,* and twelve types of sterile mycelium^25^. In China, researchers studied quinoa plants at different altitudes, the authors reported Ascomycota like the more abundant phylum in the rhizosphere and root endophytes followed by Basidiomycota, Zygomycota, and Chytridiomycota among which it was found that *Penicillium* being the most abundant fungus; in addition, they concluded that the roots presented mainly endophytic microorganisms present in the rhizosphere soil nevertheless the alpha-diversities increased at higher elevations where the hosts were found, however, the authors did not report the presence of *Fusarium*^26^.

How the precise identification of *Fusarium* is problematic, is necessary to do the identification with a multigene phylogeny for which the molecular markers ITS rDNA and EF-1α can be used because these gene sequences can solve the problem of the morphological identification; in this phylogenetic characterization, isolates produced single clades; however, there were subclades, which means there may have been race differentiation^27,28^. Using these molecular markers in our research found four species corresponding to *F. oxysporm, F. graminearun, F. culmorum*, and *F. equiseti*. Stefańczyk et al*.* (2016) reported a close phylogenetic relationship between *F. culmorum*, and *F. graminearum*^29^, species that also presented phylogenetic closeness in this study.

Our results indicated that *F. graminearum* is the most virulent species in the pathogenicity model established because this induced a more severe yellowing and wilting of Piartal cultivar leaves quinoa. This species is considered one of the most destructive plant pathogens globally and is the leading cause of *Fusarium* head blight (FHB) in cereals and is also responsible for considerable economic losses in barley, oats, and rye^30^. Further, it has mycotoxigenic capacity mainly by generating mycotoxins from the trichothecene family, making it a significant threat to food security^31^. Wang et al*.* (2021) described *F. gramineaum* presented a low virulence in corn^32^, contrary to what was found in this investigation. However *F. equiseti*, presented a similar behavior since it presented a low presence.

We also report *F. culmorum* this one too has been reported as a causal agent of FHB causing root rot in multiple hosts such as wheat, barley, oats, rye, corn, sorghum, grasses, sugar beet, flax, carnation, broad bean, pea, asparagus, red clover, strawberry, potato tuber, among others^33^. This is one of the species with less virulence in this work, has been reported as one of the species that is categorized within the *Fusarium incarnatum-equiseti* species complex (FIESC), which has about 33 phylogenetic species with a wide range of species. Distribution of habitats as well as hosts worldwide. For this reason, Wang et al*.* (2019) describe the usefulness of TEF-1α, calmodulin (CAM), the largest subunit of RNA polymerase partial (RPB1) and second-largest partial RNA polymerase subunit (RPB2) as markers for assertive differentiation of FIESC species^34^.

A similar scenario occurs with *F. oxysporum*, the most predominant species in this research. This species has a group of pathotypes called the *F. oxysporum* species complex (FOSC). It can affect more than 100 species of plants, ranking among the ten pathogens that generate the most economic losses worldwide and whose genetic relationship and high specificity with the host was recently described^35^. These species have been reported as one of the main causal agents of wilt in different crops in south America; which have been considered as a phytopathogenic complex, which under favorable conditions cause the disease and cause economic losses devastating by affecting 60% to 100% of the cultivated area^36^.

In addition, alarms have been raised in the health sector because FOSC is the second most common and virulent fungus reported, generating pathologies such as keratitis, onychomycosis, dermatitis, and allergies^37,38^. Wang et al. (2020) reported that FOSC isolated human pathogens could colonize plants without causing disease; therefore, these may be silent reservoirs, increasing the risk of immunosuppressed patients coming into contact with or consuming contaminated fruits and plants; the authors suggest the possibility of gene exchange between a pathogenic strain with a non-pathogenic one, giving virulence to ubiquitous plant fungi^39^. According to Zuriegat et al*.* (2021), during the infection process, *F. oxysporum* respond to extrinsic abiotic stresses from the environment and the host and this secretes various virulence factors, such as cell wall-degrading enzymes, effectors, and mycotoxins, that potentially play important roles in fungal pathogenicity^40^. Additionally, there exists a synergistic association of *F. oxysporum* in different cultures of *F. graminearum*^36^.

Regarding the comparison of inoculation methods, in our study, we found that the disease incidence in *F. oxysporum* and *F. graminearum* depended on the method used. Lai et al*.* (2020) have shown this relationship as the severity of *Fusarium* infections depends on the pathogenicity model applied. In sugar beet, the authors tested four inoculation methods, including barley to seed, barley to root, drenching, and cutting to determine the pathogenicity of *Fusarium* isolates^41^.

Jarek et al. (2018) evaluated both inoculation methods and aggressiveness of isolates of four *Fusarium* species (*F. proliferatum, F. oxysporum, F. verticillioides, F. solani*) on peach palm. The use of *Fusarium*-colonized ground corn for root inoculation was effective and reduced the level of damage to plants. In this sense, the authors report that root inoculation with *Fusarium*-colonized ground corn was the most suitable method for evaluating the fusariosis^42^. Aditionally, Rully et al. (2008) evaluated the effectiveness of inoculation methods for *F. oxysporum* f.sp. *cubense* (*Foc*) in Abaca (*Musa textilis* Nee), according to the authors, the root wound inoculation method followed by immersing the wounded plant in *Foc* conidia suspension (10^6^ conidia/mL) for 2 h before planting was the method most effective in causing gait^43^. On the basis with Miedaner et al. (2003) for *F. graminearum* or *F. culmorum* inoculation, it´s used two inoculation methods: Spraying a spore suspension on the head (spray inoculation) and Injecting a spore suspension into individual florets (point inoculation); the authors based on a study conducted on 20 elite winter wheat cultivars from Romania, Germany, and Switzerland suggest that spray inoculation is less laborious for routine large-scale selection of breeding materials, although there seem to be no critical differences in both methods for the development of the disease^44^.

In soil, the four *Fusarium* species reported in this study affected the root development of quinoa seedlings, which can lead to deterioration and subsequent wilting of the plant. Dřímalková and Veverka (2004) previously reported *Ascochyta caulina*, *F. avenaceum, Fusarium spp., Alternaria* spp. and *Pythium* spp., as causal agents of quinoa wilting in greenhouses. According to the authors, a comparison of the reaction of quinoa with that of other susceptible plants (spinach, cabbage, sugar beet) showed that quinoa is more susceptible to the pathogen before emergence, during germination and until the first pair of true leaves appear^45^. Germinating quinoa seeds seemed to have a lower ability to emerge from the soil. Likewise, Katsunori et al. (2010) reported that damping-off caused by *Rhizoctonia* spp. and *Fusarium* spp. is one of the most threatening factors for the growth and yield of quinoa. The authors reported that in greenhouse seedlings wilting occurred from the emergence stage to the fourth leaf stage at all planting times, but not after the fourth leaf stage. According to the authors, the reason for the difference in the buffer ratio with planting time was not the difference in temperature or hours of sunshine, but the difference in precipitation. They also reported that the disease development depends on the soil moisture, so they recommend that one method of suppressing damping-off was to decrease soil moisture early in growth. It is necessary to mention that in the test carried out in this study, daily irrigation was maintained, guaranteeing constant soil moisture^46^.

In conclusion, *Fusarium* is a widely distributed genus in quinoa crops in Boyacá Colombia, in this sense, four associated species *F. culmorum, F. equiseti, F. graminearum*, and *F. oxysporum* were found. The latter is the most abundant species. Apparently, *F. graminearum* is the most virulent species of the crop for the Piartal cultivar, however, the virulence seems to be closely related to the degree of humidity. Studies are required to determine the response of quinoa depending on the variety, as well as the virulence in mixed infections since in all cases we achieved that at least two species were present per crop. This is the first characterization of *Fusarium* associated with quinoa crops in Colombia.

## Materials and methods

### Study zone

A total of 24 strains from different locations of the Department of Boyacá, Colombia (Table 3) was selected for identification. These samples were collected in the period ranging from September 2019 to December 2020.

**Table 3:** Sampling zone data.

Municipality	Crops/Location	Coordinates	High m.a.s.l	Average temperature °C
Tunja	(1) Porvenir 1	5° 31′ 06.1″ N 73° 23′ 47.1″ W	3080	14
	(2) Porvenir 2	N 5° 33′ 66.5″ W 73° 33′ 56″	3060	11
Cómbita	(1) San Onofre	N 05° 36′ 47″ W 073° 19′ 42″	2764	13.5
Cómbita	(1) San Martín	N 05° 36′ 47.4″ W 073° 19′ 40.1″	2762	13.5
Siachoque	(1) Tocavita	N 5° 30′ 0.6″ W 73° 29′ 52.6″	2778	11.2
	(2) Guatichá L1	N 5° 29.1′ 0.8″ W 73° 30′ 60″	2778	12
Tuta	(1) Hacienda	N 05° 34′ 51.9″ W 073° 10′ 07.8″	2721	11
	(2) Agua Blanca	N 5° 39′ 47.0″ W 73° 15′ 13.7″	2710	13.5
Soracá	(1) Otro lado	N 05° 33′ 22.1″ W 073° 09′ 06.8″	2710	12
Tibasosa	(1) Peña Negra	N 05° 48′ 34.4″ W 73° 00′ 50.3″	2535	12.8
Oicatá	Blanca de Jericó	N 05° 36′ 47.2″ W 073° 19′ 40.1″	2760	12.6
Chivata	(1) San Francisco	N 5° 32′ 26.2″ W 73° 14′ 14.3″	2657	11
Sotaquirá	(1) Cortadero	N 5° 45′ 54″ W 73° 14′ 53″O	2760	11

The plant samples collected in this investigation correspond to samples of non-wild agricultural crops, the samples were obtained in accordance with the relevant regulations and legislation. The sample collection permits were granted by the “Instituto de Investigación de Recursos Biológicos Alexander Von Humbolt”—Registro Único Nacional de Colecciones (RNC) of Colombia covered by the Specimens Registry in application of article 6 of Law 1955 of 2019.

### Isolation of *Fusarium* spp.

In order to verify the presence of the four *Fusarium* species in other organs and in the rhizosphere soil of diseased plants, from these, the fungi were isolated from leaf tissue, root tissue, panicles, and rhizospheric soil. The disinfected vegetal segments were placed on 1.5% water agar plates, and incubated seven days at 12–12 h light–dark condition at room temperature. In rhizospheric soil, serial dilutions were made to isolate the fungi. For this, 10 g of sifted soil was dissolved in 90 mL of sterile distilled water and mixed with a stomacher, the samples were diluted tenfold until reaching the 10–4 dilution, 0.1 mL of each dilution was spread on PDA supplementing with 0.1 g/L of chloramphenicol and incubated seven days.

### Morphology characterization

The description of the macroscopic characters and the maintenance of the monosporic cultures was carried out in PDA medium. The description of the microscopic structures was made from monosporic cultures incubated at 25 °C with a photoperiod of 12 h for 14 days in Spezieller Nahrstoffarmer agar (SNA)^47^ and carnation agar medium^48^. Each isolate was mounted in a lacto-phenol blue solution (Sigma-Aldrich, Munich, Germany), and examined under a light microscope (Olympus Cover 015, Life Science Solutions). The description of the colonies was made taking into account colony color in the front and back direction, descriptions of aerial mycelium, conidial size, shape, and conidia color of each species. Morphological characteristics of the isolate were then compared with identification guides such as Introduction to food and airborne fungi, The *Fusarium* laboratory manual and Fungi and food spoilage^19,20,49^.

### Molecular phylogeny

To confirm the identity of the fungi, Wizard® Genomic DNA Purification Kit—Promega was used for the total genomic DNA extraction. We made the extraction from mycelia previously frozen for at least 24 h and lyophilized for 48–72 h with a lyophilizer Freezone Plus 4.5 L Labconco® using the. The protocol was modified as follows: 200–500 mg of lyophilized mycelium were taken and 600 μL of solution for nuclear lysis were added and incubated at 65 °C for 15 min, then the manufacturer's instructions for extracting DNA plant tissue purification were followed.

The molecular identification was carried out from two gene regions, ITS and EF-1α through polymerase chain reaction (PCR). The gene regions were amplified with ITS1 (5′-TCCGTAGGTGAACCTGCGG-3′) and ITS4 (5′-TCCTCCGCTTATTGATATGC3-3′) and EF1-983F (5′-GCYCCYGGHCAYCGTGAYTTYAT) and EF1-2218R (5′-ATGACACCRACRGCRACRGTYTG) primers^50,51^. For de ITS primers the PCR amplification was carried out using an Axygen® MaxyGene™ thermocycler (Corner Laboratories). It was carried out by modifying the PCR conditions proposed by Kumar and Shukla (2005) as follows: The 25 µL reaction mix contained 100 µM dNTPs, 0.1 µM of each primer, 12.5 µL 2 × PCR Taq Master mix G013 abm®, 2 µL of template DNA sample (in a concentration range of 20 to 100 ng/µL). The reaction involved initial denaturation at 94 °C for 3 min, followed by 35 serial cycles of denaturation at 95 °C for 1 min, hybridization at 56 °C for 30 s, and extension at 72 °C for 1 min, with a final extension of a cycle at 72 °C for 7 min^52^.

For identification by PCR with elongation factor-1α, the modified protocol of O’Donnell et al. (2022) was implemented^53^. The reaction was prepared for a final volume of 30 uL, which contained 15 μL of 2 × PCR Taq Master mix G013 abm®, 1.2 μL of each primer (10 nM), 2 μL of DNA, and 10.6 μL of molecular biology grade water. The reaction involved initial denaturation at 95 °C for 5 min, followed by 30 serial cycles of denaturation at 94 °C for 1 min, hybridization at 60.05 °C 50 s, and extension at 72 °C for 1 min, with a final extension of a cycle at 72 °C for 3 min.

All PCR products were sequenced in both directions by a commercial sequencing service provider (Gencore, Universidad de Los Andes, Colombia). The search for sequences was carried out using the Basic Local Alignment Search Tool (BLASTn) using the National Center for Biotechnology Information (NCBI) database (http://www.ncbi.nlm.nih.gov). For phylogenetic analysis, sequences were aligned using the Mega Align tool, and the ends of the low-quality sequences were removed manually using this same program. The sequence analysis was performed using MEGA X software to construct neighbor-joining phylogenetic trees.

All samples were deposited in the collection UBCHM (Colección de Hongos y microorganismos de la Universidad de Boyacá).

### Koch's postulates

Leaves with chlorotic and necrotic spots were collected from each crop to determine the pathogenic character of the *Fusarium* isolates according to the protocol proposed by Schuck et al.^22^. Leaves were washed as previously reported to remove surface contaminants^18,54^.These leaves were placed at room temperature for seven days in humid chambers to stimulate mycelium growth. The correlation of the symptoms was made by comparing the growth of *Fusarium* with the lesions found, this was done in at least 20 leaves for each crop^20^. Once the colonies corresponding to the four *Fusarium* species found (*F. culmorum*, *F. oxysporum*, *F. graminearum* and *F. equiseti*) had been purified, Koch's postulates were applied. Leaves obtained from healthy 60-day-old seedlings were inoculated on the abaxial side, with a 1 mm^2^ segment of PDA agar containing the 7-day-old colonies of each *Fusarium* species. Once inoculated, the leaves were incubated in humid chambers at 25 °C with a photoperiod of 12 h to observe the symptoms after 5 days. As a control, a segment of agar without inoculum was placed. The confirmation of species according to Koch's postulate, was carried out according to the protocol proposed by Zuriegat et al.^40^.

### Pathogenicity test in plants

To evaluate the pathogenicity of the strains, two protocols were tested. At first, the methodology proposed by Pal & Testen^55^ was followed with some modifications. Two months old quinoa saddling’s Piartal cultivar were inoculated according to the authors and kept in greenhouse conditions with an average humidity of 43% and an average temperature of 20 °C.

The second method was modified from the reports of Elwood et al. and Nalam et al.^56,57^. For each strain and control, the fungal mash was prepared as previously described, subsequently, a brush was moistened with the macerate and a sweep was carried out on the abaxial face of ten leaves of each two-month-old quinoa plant, the procedure was carried out three times for each leaf. Subsequently, maintaining an average humidity of 50% and an average temperature of 21 °C. In both methods, for each strain and control to be tested, ten plants were inoculated and symptom development was assessed over three weeks. For means comparison, the data obtained were processed by simple analysis of variance and T- test, with a confidence level of 95% (*p* ≤ 0.05).

### Pathogenicity test in inoculated soil

To evaluate the pathogenicity in soil, we modified the protocol proposed by Khan et al.^58^ and we inoculated germination trays with 1 mL of a suspension of conidia of each species at a concentration of 1 × 10^6^ conidia/mL, the soils were left to incubate for 24 h at 27 °C. Subsequently, previously disinfected seeds were sown (as previously mentioned for leaves), and daily irrigation was maintained, guaranteeing constant soil moisture. The soil temperature was: 19 °C. The trays were kept in greenhouse conditions with an average humidity of 43% and an average environmental temperature of 20 ± 2 °C, with daylight conditions. After 10 days of growth, the physiological indices corresponding to total length, root length and fresh mass were evaluated^59^. For this, 20 seedlings that presented the development of the first two leaves were randomly taken. Assays were performed in triplicate and data were analyzed as previously mentioned.

## Data Availability

The data obtained in this article were the product of the research carried out, data were not taken from databases or previous research. All sequences produced in this study are publicly available in NCBI GenBank Database, https://www.ncbi.nlm.nih.gov/genbank/. Fungal isolates are available in the “Colección de Hongos y Microorganismos de la Universidad de Boyacá”, UBCHM. The datasets used and/or analyzed during the current study available from the corresponding author on reasonable request. All data generated or analyzed during this study are included in this published article.

## References

[1] Dongzhen F, Xilin L, Xiaorong C, Wenwu Y, Yunlu H, Yi C (2020). *Fusarium* species and *Fusarium oxysporum* Species Complex genotypes associated with Yam Wilt in South-Central China. Front Microbiol..

[2] Chandra-Nayaka S, Wulff EG, Udayashankar AC, Nandini BP, Niranjana SR, Mortensen CN (2011). Prospects of molecular markers in *Fusarium* species diversity. Appl. Microbiol. Biotechnol..

[3] Bansal Y, Singla N, Kaistha N, Sood S, Chander J (2019). Molecular identification of *Fusarium* species complex isolated from clinical samples and its antifungal susceptibility patterns. Curr. Med. Mycol..

[4] Bertero A, Moretti A, Spicer LJ, Caloni F (2018). *Fusarium* molds and mycotoxins: Potential species-specific effects. Toxins (Basel)..

[5] Zhang Y. & Ma L. J. Deciphering Pathogenicity of *Fusarium oxysporum* From a Phylogenomics Perspective. In *Advances in Genetics*. 1st Edn. 179–209 (Elsevier Inc., 2017). 10.1016/bs.adgen.2017.09.010 10.1016/bs.adgen.2017.09.010 29153400

[6] Ryabova N, Tupolskikh T, Serdyuk V, Gordeeva N (2021). Analysis of infection with fungi of the genus *Fusarium* seed and vegetative organs of crops. E3S Web Conf..

[7] Arie T (2019). *Fusarium* diseases of cultivated plants, control, diagnosis, and molecular and genetic studies. J. Pestic. Sci..

[8] Palacios SA, Del Canto A, Erazo J, Torres AM (2021). *Fusarium cerealis* causing *Fusarium* head blight of durum wheat and its associated mycotoxins. Int. J. Food Microbiol..

[9] Pessôa MG, Paulino BN, Mano MCR, Neri-Numa IA, Molina G, Pastore GM (2017). *Fusarium* species—a promising tool box for industrial biotechnology. Appl. Microbiol. Biotechnol..

[10] De Bock P, Van Bockstaele F, Muylle H, Quataert P, Vermeir P, Eeckhout M (2021). Yield and nutritional characterization of thirteen quinoa (*Chenopodium quinoa* willd.) varieties grown in north-west europe—part i. Plants..

[11] Sobota A, Świeca M, Gęsiński K, Wirkijowska A, Bochnak J (2020). Yellow-coated quinoa (*Chenopodium quinoa* Willd): Physicochemical, nutritional, and antioxidant roperties. J. Sci. Food Agric..

[12] Urbina H, Breed MF, Zhao W, Lakshmi-Gurrala K, Andersson SGE, Ågren J (2018). Specificity in *Arabidopsis thaliana* recruitment of root fungal communities from soil and rhizosphere. Fungal Biol..

[13] Bouras H, Choukr-Allah R, Amouaouch Y, Bouaziz A, Devkota KP, El Mouttaqi A (2022). How does quinoa (*Chenopodium quinoa* Willd.) respond to phosphorus fertilization and irrigation water salinity?. Plants..

[14] Villacrés E, Quelal M, Galarza S, Iza D, Silva E (2022). Nutritional value and bioactive compounds of leaves and grains from quinoa (*Chenopodium quinoa* Willd.). Plants..

[15] Danielsen S, Munk L (2004). Evaluation of disease assessment methods in quinoa for their ability to predict yield loss caused by downy mildew. Crop Prot..

[16] Drimalkova M-VK (2003). Seedlings Damping-off of Chenopodium quinoa Willd. Plant Prot..

[17] Testen AL, Jiménez-Gasco MDM, Ochoa JB, Backman PA (2014). Molecular detection of *Peronospora variabilis* in quinoa seed and phylogeny of the quinoa downy mildew pathogen in South America and the United States. Phytopathology.

[18] Fonseca-Guerra I, Chiquillo-Pompeyo C, Padilla MJ, Benavides-Rozo M (2021). First report of bacterial leaf spot on *Chenopodium quinoa* caused by *Pseudomonas syringae* in Colombia. J. Plant Dis. Prot..

[19] Leislie J. & Summerell B. *The Fusarium laboratory manual*. 1st edn, 1–388 (Blackwell Publishing, 2006). http://ir.obihiro.ac.jp/dspace/handle/10322/3933

[20] Pitt JI, Hocking AD (2009). Fungi and Food Spoilage.

[21] Smith JA, O’Donnell K, Mount LL, Shin K, Peacock K, Trulock A (2011). A novel *Fusarium* species causes a canker disease of the critically endangered conifer, Torreya taxifolia. Plant Dis..

[22] Schuck S, Weinhold A, Luu VT, Baldwin IT (2014). Isolating Fungal Pathogens from a Dynamic Disease Outbreak in a Native Plant Population to Establish Plant-Pathogen Bioassays for the Ecological Model Plant Nicotiana attenuata. PLoS ONE.

[23] Farahani-Kofoet RD, Witzel K, Graefe J, Grosch R, Zrenner R (2020). Species-specific impact of *Fusarium* infection on the root and shoot characteristics of asparagus. Pathogens..

[24] González-Teuber M, Vilo C, Bascuñán-Godoy L (2017). Molecular characterization of endophytic fungi associated with the roots of *Chenopodium quinoa* inhabiting the Atacama Desert, Chile. Genomics Data..

[25] Aletaha R, Sinegani AAS, Zafari D (2018). A survey on endophytic fungi within roots of Chenopodiaceae species under different environmental conditions. Mycosphere.

[26] Cai Z, Wang X, Bhadra S, Gao Q (2020). Distinct factors drive the assembly of quinoa-associated microbiomes along elevation. Plant Soil..

[27] Paul NC, Park W, Lee S, Chung MN, Lee HU, Yang JW (2020). Occurrence of sweetpotato (*Ipomoea batatas*) wilt and surface rot disease and determining resistance of selected varieties to the pathogen in Korea. Plants..

[28] Summerell BA (2019). Resolving *Fusarium* : Current Status of the Genus. Annu. Rev. Phytopathol..

[29] Stefańczyk E, Sobkowiak S, Brylińska M, Śliwka J (2016). Diversity of *Fusarium* spp. associated with dry rot of potato tubers in Poland. Eur. J. Plant Pathol..

[30] Mentges M, Glasenapp A, Boenisch M, Malz S, Henrissat B, Frandsen RJN (2020). Infection cushions of *Fusarium graminearum* are fungal arsenals for wheat infection. Mol. Plant Pathol..

[31] Ducos C, Pinson-Gadais L, Chereau S, Richard-Forget F, Vásquez-Ocmín P, Cerapio JP (2021). Natural occurrence of mycotoxin-producing fusaria in market-bought Peruvian cereals: A food safety threat for Andean populations. Toxins (Basel)..

[32] Wang W, Wang B, Sun X, Qi X, Zhao C, Chang X (2021). Symptoms and pathogens diversity of Corn *Fusarium* sheath rot in Sichuan Province, China. Sci. Rep..

[33] Scherm B, Balmas V, Spanu F, Pani G, Delogu G, Pasquali M (2013). *Fusarium culmorum*: Causal agent of foot and root rot and head blight on wheat. Mol. Plant Pathol..

[34] Wang MM, Chen Q, Diao YZ, Duan WJ, Cai L (2019). *Fusarium incarnatum-equiseti* complex from China. Persoonia Mol. Phylogeny Evol. Fungi..

[35] Zhang Y, Ma LJ, Townsend JP, Wang Z (2017). Deciphering Pathogenicity of *Fusarium oxysporum* From a Phylogenomics Perspective. Advances in Genetics.

[36] Villa-Martínez A, Pérez-Leal R, Morales-Morales HA, Ba-Surto-Sotelo M, Soto-Parra JM, Martínez-Escudero E (2014). Current situation of *Fusarium* spp in the control and evaluation of the an-tifungal activity on vegetables extracts. Acta Agron..

[37] Batista BG, de Chaves MA, Reginatto P, Saraiva OJ, Fuentefria AM (2020). Human fusariosis: An emerging infection that is difficult to treat. Rev. Soc. Bras. Med. Trop..

[38] Veiga FF, De Castro-Hoshino LV, Sato F, Bombassaro A, Vicente VA, Mendes V (2018). *Fusarium oxysporum* is an onychomycosis etiopathogenic agent. Fut. Microbiol..

[39] Wang CJ, Thanarut C, Sun PL, Chung WH (2020). Colonization of human opportunistic *Fusarium oxysporum* (HOFo) isolates in tomato and cucumber tissues assessed by a specific molecular marker. PLoS ONE.

[40] Zuriegat Q, Zheng Y, Liu H, Wang Z, Yun Y (2021). Current progress on pathogenicity-related transcription factors in *Fusarium oxysporum*. Mol. Plant Pathol..

[41] Lai X, Qi A, Liu Y, Del Río Mendoza LE, Liu Z, Lin Z (2020). Evaluating inoculation methods to infect sugar beet with *Fusarium oxysporum* f. Betae and *F*. *secorum*. Plant Dis..

[42] Jarek TM, dos Santos ÁF, Tessmann DJ, Vieira ESN (2018). Inoculation methods and aggressiveness of five *Fusarium* species against peach palm. Cienc Rural..

[43] Rully DP, Nurul H, Sudjindro S (2008). Inoculation Methods and conidial densities of *Fusarium oxysporum* f.sp. cubense in Abaca. HAYATI J. Biosci..

[44] Miedaner T, Moldovan M, Ittu M (2003). Comparison of spray and point inoculation to assess resistance to *Fusarium* head blight in a multienvironment wheat trial. Phytopathology.

[45] Dřímalková M, Veverka K (2010). Seedlings damping-off of Chenopodium quinoa Willd. Plant Prot. Sci..

[46] Isobe K, Sugiyama T, Katagiri M, Ishizuka C, Tamura Y, Higo M, Fujita Y (2019). Study on the Cause Damping-off in Quinoa (Chenopodium quinoa Willd) and a Method for Suppressing its Occurrenceキノアの立枯れの発生原因と抑制法に関する研究. Jpn. J. Crop Sci..

[47] Nirenberg HI (1976). Untersuchungen iiber die morphologische und biologische Differenziemng in der *Fusarium* SektionLiseola. Mitt. Biol. Bundesanst. LandForstwirtsch. Berlin-Dahlem..

[48] Fisher N, Burgess L, Toussoun T, Nelson P (1982). Carnation leaves as a substrate and for preserving cultures of *Fusarium* species. Phytopathology.

[49] Samson R. A, Hoekstra E. S. & Frisvad J. C. Introduction to food- and airborne fungi (ed Samson R.) 7th edn. 1–389 (Amer Society for Microbiology, 2004).

[50] White T. J., Bruns T., Lee S. & Taylor J. Amplification and direct sequencing of fungal Ribosomal Rna Genes for phylogenetics. In: *PCR Protocols*. 1st edn, 315–22 (Academic Press Inc., 1990).

[51] Rehner SA, Buckley E (2005). A Beauveria phylogeny inferred from nuclear ITS and EF1-α sequences: Evidence for cryptic diversification and links to Cordyceps teleomorphs. Mycologia.

[52] Kumar M, Shukla PK (2005). Use of PCR targeting of internal transcribed spacer regions and single-stranded conformation polymorphism analysis of sequence variation in different regions of rRNA genes in fungi for rapid diagnosis of mycotic keratitis. J. Clin. Microbiol..

[53] O’Donnell K, Laraba I, Geiser DM, Coleman J (2022). Pure Culture and DNA Sequence-Based Identification of *Fusarium* from Symptomatic Plants and Diverse Substrates. Fusarium wilt Methods in Molecular Biology.

[54] Pitzschke A (2016). Developmental peculiarities and seed-borne endophytes in Quinoa: Omnipresent, robust bacilli contribute to plant fitness. Front Microbiol..

[55] Pal N, Testen AL (2020). First report of quinoa anthracnose caused by *Colletotrichum nigrum* and *C. truncatum* in the United States. Plant Dis..

[56] Nalam V, Sujon S, Jyoti S (2016). Establishment of a *Fusarium graminearum* Infection Model in *Arabidopsis thaliana* Leaves and Floral. Bio-Protoc..

[57] Ellwood S, Kamphuis L, Pfaff T, Oliver R, Foster-hartnett BD, Villegas AM (2007). Inoculation and growth with foliar pathogenic fungi. Medicago truncatula Handb..

[58] Khan MF, Bhuyian Z, Lashman D, Liu Y, Mosher P, Knoke S (2022). First report of damping off and seedling rot of hemp (*Cannabis sativa* L.) caused by *Fusarium solani* (Mart.) Sacc. in North Dakota, USA. Plant Dis..

[59] Abdelmagid A, Hafez M, Lawley Y, Adam LR, Daayf F (2018). First report of *Fusarium cerealis* causing root rot on soybean. Plant Dis..

